# Observations on the Functions of the Liver, and on the Importance of That Organ as Possessing Great Associate Influence, and as a Frequent Seat of Morbid Affections; Being an Abstract of a Paper Read before the Physical Society of New York, October 7, 1802

**Published:** 1808-10

**Authors:** Malachi Foot


### OBSERVATIONS *on the* FUNCTIONS *of the* LIVER, *and on the* IMPORTANCE *of that* ORGAN *as possessing great* ASSOCIATE INFLUENCE, *and as a frequent* SEAT *of* MORBID AFFECTIONS; *being an Abstract of a Paper read before the Physical Society of New York, October 7, 1802. By Dr.* MALACHI FOOT.

A CONSIDERATION of the *associate* diseases of the liver would be naturally preceded by that of the *primary* diseases of that organ, and also a description of those morbid conditions of the alimentary canal, arising from the varying states of the bilious secretion; but a detail so lengthy as would be necessary fully to accomplish this, is, with propriety, here limited to a few observations.

The liver, whether we form our estimate from its functions in health, or the evil consequences of its diseased state, is one of the most important organs of the human body. Its office, together with those of the *gall-bladder* and *ductus communis choledochus,* is to supply the intestines with bile for the purposes of digestion, and preventing the inconveniencies resulting from the natural decomposition of food within the alimentary canal.

Digestion has, till lately, been considered as one of the most mysterious functions of the animal body. It is the great leading process of that assimilation which converts dead animal and vegetable matter into nutriment and organized life.

Digestion differs so materially from that natural decomposition of bodies, during the process of fermentation, that the slightest degree of the latter, within the alimentary canal, is productive of inconvenience. The disengagement of noxious gases, and especially of that mephitic poison, a product of the putrefactive process, abounding particularly in the lean of animal meats, is among the evils of imperfect assimilation.

The great efficacy which the bile exerts in preventing these evils is not always justly appreciated. The properties of pure bile are saponaceous, alkaline, and oleaginous; whence the liver has lately, with much propriety, been termed "the great manufactory of soda for the purposes of the system."

Whether *dyspepsia* is ever idiopathic, simply as respects the stomach, is very problematical. The old opinion, that trituration constitutes a very considerable part of the business of digestion, has become more inadmissible since the the explosion of mechanical doctrines as explanatory of living action.* The gastric juice being a secreted fluid, like all other secretions, varies with the state of its secretory organ; yet the marks of diversity, as to its quantity or morbid qualities, are by no means so conspicuous and distinct as those of the *bilious* secretion.†

There exists between the stomach and liver a most intimate and important association. The latter, in its healthy state, is ever ready to perform its offices at the instance of the former. In ordinary states of the digestive function, when the articles taken in are of a proper nature, and in due quantity, the presence of a very large store of bile is not necessary; but such, is its importance, that an absence of this friendly fluid, even for a short time, merely as respects the stomach, is attended with the greatest inconveniences. Its primary and ordinary office is to meet the aliment in its passage from the pylorus through the duodenum, and at that instant when it might otherwise assume the most noxious form, intimately to combine with it, disarm it of its deadly weapons, and render it a peaceable passenger in its mazy course. The quantity of bile excreted into the duodenum, within certain bounds, is proportionate to its need. Such is the animal economy, that those qualities which require an extraordinary quantity, provoke an unusual secretion. This is particularly exemplified by the presence in the stomach of a strong acid, and especially that noxious form of it resulting from the natural decomposition of aliment. When united to an acid, bile is changed to a greenish colour, termed "porraceous," and, in this vitiated and remarkable form, is exposed to our view. Such are the healthful qualities of this fluid, in its pure state, that the term "redundancy of bile," in Physic, is as improper as that of "excessive virtue," in Ethics.

Do the several modifications of diseases of the alimentary canal arise from diversity of temperaments (as they are described by Dr. Darwin) and the influence of epidemic causes? May not the liver often be said to be the primary seat

---

* See the Experiments of Spallanzani on this subject.

† Dispensing with the consideration of the theory of digestion, every physician must have noticed, that the most prominent symptoms attending the dyspeptic may be resolved into the effects which naturally result from a deficiency in the bilious secretion. Does habitual dyspepsia seldom occur without evident marks of a *torpid liver*?

seat of these diseases, or the medium through which epidemic causes operate? And will not a balance between the noxious agents and the antiseptic virtues of pure bile ever insure health? Inferences, drawn from some modern principles, promise much towards a solution of these inquiries.

Sympathy may be defined to be that synchronous or successive condition of two or more parts of living bodies, arising from the operation of a local cause. Mechanic reasoning can with no propriety be applied to the explanation of the important doctrines of association. It is not satisfactorily explained by the trite notions of vascular or nervous anastomoses; nor is it to be assigned, *en masse*, to contiguity of origin in the sensorium commune. To become an adept in this science, requires a more complete acquaintance with that principle which constitutes the difference between living and dead matter, the origin of our early motions, and the manner and extent of the operation of external agents. Though I cannot second the declaration of the great author of the Brunonian system in execrating physiology, yet I possess a strong impression that the study of it contributes but partially to an acquaintance with the interesting doctrines of morbid association. As a specimen of its inadequacy, I might challenge the ingenuity of the best informed physioligist, even with the aid of a most lively fancy, to explain satisfactorily the phenomenon of *singultus nephriticus* (hiccup from calculus in the kidney), or the cause of *tensio penis* in *hydrophobia.*

The important office of the liver, in furnishing the bile, is only equalled by its possessing over the system a stroug and most extensive associate influence. This organ obviously derives its extensively controuling powers through the medium of the stomach, which having commenced its functions in our earliest embryo-state, and being the great seat of our pleasurable sensations, may almost be termed the seat of life.

It is a happy provision in the animal economy, that these organs, exposed, as they are, from the nature of their offices, to the exciting causes of disease, while they possess a high degree of *irritability*, are almost destitute of *sensibility*. The liver, as a second link of a train of associations, and possessing rather more sensibility than its principal (the stomach), assumes her morbid states, and transfers them to a third part, possessed of still more than herself: thus gastritis is a disease of less frequent occurrence than hepatitis, and a phlegmon of an inferior extremity, tremity, or an affection of the parts about the right shoulder, is of more frequent occurrence than either.

Among the associate diseases of the liver we may class some of the following.

1. *Erysipelas.* This is a sensitive affection of the external surface, frequently sympathetic of a morbid irritation of the membranes about the liver, and belongs to Dr. Darwin's class of associate diseases. As there is an intimate consent between the internal viscera and external surface generally, this disease may arise from an affection of some other viscus. It is commonly preceded by a fever of two or three days continuance, terminating in this metastasis (of action, not of fluid) to the surface. As the cause does not reside in the place of its appearance, it is liable to recede on exposing the part to cold. Of the same origin is the pimpled face of females of habits of great associability, and the *gutta rosea* of intemperate persons.

2. *Gout* and *Rheumatism.* Are not these diseases often of hepatic origin.

3. *Colica Saturnina.* The *modus operandi* by which *lead* produces its deleterious effects is not well understood. Its most obvious property is that of a great stimulus to the absorbent system. In the cases in which it proves injurious, marks of a torpid liver are conspicuous. The general symptoms are, dyspepsia, flatulence, costiveness, colic pain, pallid complexion, low spirits, a partial paralyis, more especially of the right arm and shoulder, sometimes attended with a severe pain—hepatic cough, and general debility. A removal of the usual symptoms, in a case of this kind, by mercury, was succeeded by a permanent *gutta rosea* of the face.

4. *Asthma.* Of the two species of this disease, the humid is often a sympathetic affection. The lungs and liver, separated only by the diaphragm, are intimately associated. The exciting cause of humid asthma is the irritation of a saline mucus lodged in the air cells from deficient absorption. In the case of *torpor hepaticus* it may arise from *direct* sympathy; in *chronic hepatitis* from *reverse.* The laborious respiration and cough are a combination of voluntary and sensitive motion, for the purpose of relieving pain and removing the offending cause. For the sake of illustration, I trust I shall be excused in relating briefly the history of a case most obviously apposite.

Among other marks of an occasional occurrence of chronic hepatitis, or a morbid degree of irritation of the membranes about the liver, the most troublesome was an obtuse obtuse pain in the right hypochondre, attended with much anxiety. This succeeded an attack of intermittent fever, during a short residence in a marshy country. The first and most conspicuous associate symptom was a periodical head-ach, generally in the form of hemicrania; commencing with cold chills, and, for the most part, terminating in anorexia and vomiting. This translation of pain was attended with a removal of all sensitive affection from the primary part, and was obviously owing to an increased sensibility and mobility of the membranes about the head, produced by a change of employment from an easy and sedentary life, to the situation of a person constantly exposed to all the vicissitudes of temperature between the extremes of heat and cold. A removal of these causes, by a change of residence, and the inexposure and ease of a city life, with the inconvenience of a frequent inhalation of deleterious gases, has converted this form of disease to a most troublesome *asthma*. That this form of affection results from the original cause (morbid irritation of the hepatic membranes), is rendered probable both by the circumstances attending the occurrence of the paroxysms, and the operation of remedies. That, in this instance, a reverse sympathy obtains between the liver and lungs, is rendered conclusive by the two-fold consideration, that both those causes which produced a direct influence on the former, through the medium of the stomach—as the stimulus of a full meal, acids, aromatics, or wine—as readily and invariably produce its paroxysms as the direct operation of noxious gases on the *lungs*, or the sudden exposure of that organ, and also the surface, to a cold and moist atmosphere. Correspondent to the operation of the exciting causes is also the operation of remedies. A diminished excitement of the stomach and liver, by the action of digitalis— a nauseating portion of ipecacuanha—or fifteen hours abstinence—are not less certain in procuring relief than the inhalation of the fumes of tobacco— breathing the warm and dry air of a tight room—or the stimulus of additional flannel to the surface. The most complete relief from this very uncomfortable disease has, of late, been obtained, by taking three grains of *mild muriate of quicksilver* for eight successive days. Does the certain temporary relief derived from the high diffusible stimuli, as opium and camphor, arise from the universality of their operation in dissevering morbid associations, and exciting increased action throughout the system?

5. *Phlegmon of the extremities*, without evident local cause, cause—that species of affection termed *fever-sore—paronychia* and *boils.* Are these frequently substitutes for acute inflammation of the liver, or sympathetic of a chronic disease of that viscus?

6. *Sphacelation of the fleshy parts,* with a caries of the bones of the toes. This curious species of affection, though not of frequent occurrence, is, however, under the usual mode of treatment, as a local disease only, often very troublesome, extending to the metatarsal bones, and gradually wasting the whole foot. Permit me to state, that two cases of this kind, which have come under my inspection, have afforded ample proofs of their hepatic origin. Does the known sympathy between the liver and feet place this affection, as to origin, on the same ground of probability with *gout?* and does the benefit arising from a liberal use of opium in these cases, as recommended by Mr. Pott, result from its high stimulant powers in exciting the healthy actions of a torpid liver?

Among errors in nosology, probably there are none more universal than the opinion that certain symptoms, expressive of hepatic disease in children, denote the existence of worms in the alimentary canal. That those symptoms frequently indicate their existence is well ascertained; but in a majority of cases, they unequivocally arise from a want of the healthy actions of the liver, of which the existence of worms is only a symptom.

I shall conclude this part of my subject by stating the intimate connection between our mental faculties and enjoyments, and a healthy state of this organ.

The two great sources of our happiness are the exercise of our intellects, and those healthy operations of our bodies of which we are unconscious, and which, unlike the former, commence with our existence. Reasoning *a posteriori,* we may safely conclude that a very considerable number of the cases of *tædium vitæ* arise from a disordered state of this organ. Our pleasurable and painful sensations so uniformly correspond with the healthy and morbid states of the liver, that they reciprocally excite each other. So sensible of this connection were the ancient writers on medicine, that, by the use of a simple term to express either, they conveyed the complex idea of both: hence the word *melancholia,* from the Greek μελας, *black,* and χολη, *bile*; hence also the term *hypochondriasis,* referring to that region of the abdomen which is the seat of the disease; and when they described a person of an irritable and and peevish disposition, they termed him *choleric*, from χολερα, a diseased state of the bile.

Among the causes of hepatic diseases we may count some of the following.

1. Intemperance in the use of spirituous liquors, and other high diffusible stimuli.

2. Intense and constant grief; and a sedentary life, with close study on subjects destitute of amusement.

3. The constant practice of equitation in rough countries; and the swallowing large quantities of cold liquids, with the body previously heated.

4. The practice of too frequent taking of food between the regular periods of meals, and an inordinate use of saccharine and oily food. Does the latter article produce a torpid liver, by destroying the stimulant properties of the gastric juice, from chemical operation? We are told, by the satirical Horace, in describing the capons of that day, that the Romans possessed the art of enlarging the livers of their fowls to the size of the whole body. This art is practised by the Sicilians of the present day; and it is said to be effected by feeding them liberally with food impregnated with oil.* Common practice prohibits the use of oily food in all cases of troublesome herpetic eruption, of which a great proportion are sympathetic of hepatic affections.

5. Imprudent removal of flannel dress; cold and damp lodgings; and an improper use of the cold bath.

6. Vicissitudes of weather, and change of climate. Amongst European emigrants, no people appear to experience the inconvenience of our climate more sensibly than the Welsh. What should constitute so wide a difference between that people and the Irish, I am not able satisfactorily to explain. The climate of *Wales* is not more temperate, nor the weather less variable, than in *Ireland*. Is this difference to be imputed to diversity in the modes of life, and peculiar national temperaments?

7. The operation of certain *deleterious gases*. Like the case of the clown and violin, an illustration well introduced, on another occasion, by the ingenious Dr. Rush; whenever I view this subject I am obliged to relinquish it, with the poor consolation of having only discovered the way leading to hidden treasure. Much light is thrown on it

* Vide Zoonomia, vol. i. p. 258.

it in a *Dissertation on the sympathy of the stomach.** In all cases of *fever*, whether intermittent, remittent, continued, malignant, or yellow fever, and the concomitant diseases of that situation and season most favourable to their production, there are almost invariable marks of an affection of the liver.

Those symptoms usually and improperly denominated *bilious*, and attendant on these fevers, are familiarly spoken of by physicians, without ever attempting their explanation, whether they are to be viewed in the light of cause, effect, or symptom.

As the length of this paper excludes a particular examination of remedies, permit me to notice only two of those articles which admit of more general application, viz. *mercury* and *alkalies*.

If we may be allowed to compare the effects of mercury with that inert practice which accomplishes *nothing*; if an estimation of the effective powers of an article may be drawn from its ability in arresting the shafts of death, whether he approaches with gradual, but certain aim; or, rushing on with gigantic strides, boldly and suddenly stares the unhappy victim in the face—may we not, with some degree of propriety, balance this article with almost the whole of the materia medica in common use? It has, of late, (and without an hyperbole) received the appellation of the *Sampson* among remedies.

The explanation of the *modus operandi* of alkalies, and their more general introduction into practice, especially in fevers, has presented us with a new æra in medicine.

That a doctrine so discordant with those taught by most medical writers, and differing so entirely from the habits of thinking and practice of physicians, and which, from its extensive application, must effect so important a revolution in prescription, should meet with opposition, even from men of talents, is so far from militating against its worth, that a contrary reception would excite suspicion of its futility. An attempt to vindicate this doctrine, *a priori*, would be impeaching the well-known talents of its author: nor need I here assert that it is bottomed on the interesting discovery, that a gaseous poison—an oxyd—probably the compound of *septon* and *oxygen*—is a principal agent in the production of pestilence and its kindred diseases

* Vide Medical Repository, vol. v. p. 300.

diseases. The uniform nature of all the phenomena which constantly meet the eye, and are strikingly exhibited to the notice of every discerning mind, especially in epidemic seasons, conjoined to the practical testimony of a very respectable number of physicians within this city and country, of the great value of alkalies in fever and otherwise, are sufficient to establish it against the cabals of a *host* of European opponents. Consenting even to put in competition with the uniform and striking phenomena of nature, and the most unequivocal facts, the result of chemical analysis, as exhibited by artificial management, what is the conclusion? The experiments of Davy, Fourcroy, and others (even giving full credit to their testimony), amount simply to this: that *nitrous oxyd,* a principle differing materially from the *septic poison* of Dr. Mitchill, when inhaled into the lungs from a pneumatic apparatus, possesses high, and even morbid stimulant properties; and when applied to dead animal substances, retards putrefaction. To the first of these positions, even granting the identity of the agents, I shall only state, that the symptoms too of yellow fever uniformly evince that the morbid cause is a substance of high stimulant properties, and that the rapid tendency to disorganization is the result of a sudden exhaustion of the powers of life, constituting the indirect debility of Dr. Brown. Were it not too great a digression from this subject, I might trace the analogy between pestilence and the cases of poisoning by *arsenic,* the bites of rabid animals and venomous reptiles, where the morbid cause is known to be a stimulant of the highest grade. As to the objection arising from the antiseptic powers of nitrous acid, at this period of medical improvement, when the two sets of laws which regulate living and dead matter are no longer confounded, it merits not a serious consideration.

